# A machine-learning framework for robust and reliable prediction of short- and long-term treatment response in initially antipsychotic-naïve schizophrenia patients based on multimodal neuropsychiatric data

**DOI:** 10.1038/s41398-020-00962-8

**Published:** 2020-08-10

**Authors:** Karen S. Ambrosen, Martin W. Skjerbæk, Jonathan Foldager, Martin C. Axelsen, Nikolaj Bak, Lars Arvastson, Søren R. Christensen, Louise B. Johansen, Jayachandra M. Raghava, Bob Oranje, Egill Rostrup, Mette Ø. Nielsen, Merete Osler, Birgitte Fagerlund, Christos Pantelis, Bruce J. Kinon, Birte Y. Glenthøj, Lars K. Hansen, Bjørn H. Ebdrup

**Affiliations:** 1 Center for Neuropsychiatric Schizophrenia Research and Center for Clinical Intervention and Neuropsychiatric Schizophrenia Research, Mental Health Centre Glostrup, Copenhagen University Hospital, Glostrup, Denmark; 2 Cognitive Systems, DTU Compute, Department of Applied Mathematics and Computer Science, Technical University of Denmark, Kongens Lyngby, Denmark; 3 H. Lundbeck A/S, Valby, Denmark; 4 Danish Research Centre for Magnetic Resonance, Centre for Functional and Diagnostic Imaging and Research, Copenhagen University Hospital, Hvidovre, Denmark; 5 Department of Clinical Physiology and Nuclear Medicine, Rigshospitalet, University of Copenhagen, Glostrup, Denmark; 6 Department of Psychiatry, Brain Center Rudolf Magnus, University Medical Center Utrecht, Utrecht, The Netherlands; 7 Faculty of Health and Medical Sciences, Department of Clinical Medicine, University of Copenhagen, Copenhagen, Denmark; 8 Center for Clinical Research and Prevention, Bispebjerg and Frederiksberg Hospitals, Frederiksberg, Denmark; 9 Section for Epidemiology, Department of Public Health, University of Copenhagen, Copenhagen, Denmark; 10 Department of Psychology, University of Copenhagen, Copenhagen, Denmark; 11 Melbourne Neuropsychiatry Centre, Department of Psychiatry, University of Melbourne and Melbourne Health, Melbourne, VIC Australia; 12 Lundbeck North America, Deerfield, IL USA

**Keywords:** Predictive markers, Diagnostic markers, Schizophrenia

## Abstract

The reproducibility of machine-learning analyses in computational psychiatry is a growing concern. In a multimodal neuropsychiatric dataset of antipsychotic-naïve, first-episode schizophrenia patients, we discuss a workflow aimed at reducing bias and overfitting by invoking simulated data in the design process and analysis in two independent machine-learning approaches, one based on a single algorithm and the other incorporating an ensemble of algorithms. We aimed to (1) classify patients from controls to establish the framework, (2) predict short- and long-term treatment response, and (3) validate the methodological framework. We included 138 antipsychotic-naïve, first-episode schizophrenia patients with data on psychopathology, cognition, electrophysiology, and structural magnetic resonance imaging (MRI). Perinatal data and long-term outcome measures were obtained from Danish registers. Short-term treatment response was defined as change in Positive And Negative Syndrome Score (PANSS) after the initial antipsychotic treatment period. Baseline diagnostic classification algorithms also included data from 151 matched controls. Both approaches significantly classified patients from healthy controls with a balanced accuracy of 63.8% and 64.2%, respectively. Post-hoc analyses showed that the classification primarily was driven by the cognitive data. Neither approach predicted short- nor long-term treatment response. Validation of the framework showed that choice of algorithm and parameter settings in the real data was successfully guided by results from the simulated data. In conclusion, this novel approach holds promise as an important step to minimize bias and obtain reliable results with modest sample sizes when independent replication samples are not available.

## Introduction

Schizophrenia is a severe and heterogeneous brain disorder. Patients exhibit a great variety of symptoms, which span in severity from barely noticeable to completely dominating the patient’s mental state and behavior. Correspondingly, the course of illness varies from symptomatic recovery to treatment resistance with marked impairments in social functioning. Approximately half of all schizophrenia patients do not respond adequately to current antipsychotic medication, and estimates of treatment resistance vary greatly (14−60%)^1,2^. The large variability in biological measures and clinical manifestations of schizophrenia has complicated estimations of an individual patient’s prognosis.

Already at the onset of disease, schizophrenia patients display abnormalities in neuroanatomical^3,4^, electrophysiological^5,6^, and cognitive measures^7^. Changes are subtle and only apparent on the group level, and antipsychotic medication as well as duration of illness are potential confounders^8,9^. Antipsychotic-naïve patients are challenging to recruit, and most studies in antipsychotic-naïve schizophrenia patients are relatively small, clinically heterogenous, and apply a limited number of modalities.

Machine learning (ML) is a powerful computational approach to unravel patterns in complex, multivariate datasets. Emerging ML studies based on neuroimaging data have successfully classified schizophrenia patients from healthy controls and, to some degree, predicted outcome^10,11^. However, replicability of clinical findings has been challenging, and it is increasingly recognized that rigorous methodology is crucial to reduce bias and overestimations^12–14^.

In recent years, advances have been made towards combining data from multiple modalities in order to improve prediction. Clinical studies applying multimodal approaches are scarce, but may improve classification of schizophrenia patients from healthy controls compared to unimodal approaches^15^, although findings are equivocal^16^. We recently reported that the treatment response of psychopathologically indistinguishable patient subgroups was significantly predicted by an ML model based on cognitive and electrophysiological data^17^.

In the current study, we expand on our previous approach^17^ by including additional modalities and pooling data from several comparable cohorts of antipsychotic-naïve, first-episode schizophrenia patients. Different ML algorithms have varying predictive capabilities when applied to different tasks and different types of data^18^. Some investigators may only have tested one arbitrarily chosen model^10,14^, or may not have reported all tested models, thereby increasing the possibility of a type 1 error. To minimize bias in algorithm selection and parameter settings, we selected algorithms for the analysis on our real data based on their performance on simulated datasets. This novel approach reduces the risk of overfitting and provides transparency in the algorithm selection process. Furthermore, we thoroughly describe our pipeline to enable reproducibility and detail how we avoided data leakage from the training set to the test set.

Robustness of results was ensured by running two independent ML approaches in parallel. One ML approach was a conventional learning approach, using a single carefully chosen and optimized ML algorithm, and the other was a more flexible approach, which allowed multiple algorithms to be combined in an ensemble. As input data we used cognitive, electrophysiological, brain structural, and psychopathological data, as well as perinatal register data. In order to establish the framework, we aimed to predict diagnostic status. Furthermore, we aimed to predict short- and long-term treatment response. We hypothesized that our setup applied on baseline data would be able to significantly classify schizophrenia patients from healthy controls. Furthermore, we hypothesized that ML models based on multimodal data would be superior to unimodal models at predicting treatment response. Finally, we validated our methodological framework by testing if the ranking of algorithm performance on the simulated data was maintained in the real data.

## Methods

### Participants and interventions

All included patients were antipsychotic-naïve and experiencing their first episode of psychosis. Patients were recruited from in- and outpatient clinics in the Capital Region of Copenhagen, Denmark. Patients were recruited as part of three comparable, consecutive cohorts (cohorts A (1998–2002), B (2004–2008), and C (2008–2014)) (Table 1). Results from previous studies on these cohorts have been published elsewhere (e.g. refs. ^16,19,20^), and a complete list of publications is provided at www.cinsr.dk. Patients in cohort A had been randomized to treatment with either risperidone or zuclopenthixol for 3 months. In cohort B patients received treatment with quetiapine for 6 months. In cohort C patients were treated with amisulpride for 6 weeks. In all three cohorts, medication dosage was increased until a clinical antipsychotic effect was evident, while taking side effects into account.

**Table 1:** Demographic and clinical characteristics of patients with schizophrenia and healthy control subjects.

	Schizophrenia patients		Healthy controls		Statistics	*p*
	*N*	Distribution	*N*	Distribution		
Subjects, cohorts A/B/C^a^	138	31/46/61	151	27/53/71	*χ*^2^ = 0.95	0.623
Age, years, Mean (SD)^b^	135	25.36 (5.88)	146	25.48 (5.61)	*U* = 9535	0.638
Gender, Male/Female^a^	138	94/44	151	99/52	*χ*^2^ = 0.21	0.645
P-SES, High/Moderate/Low^a^	134	39/73/22	146	61/70/15	*χ*^2^ = 5.72	0.057
Years of education, Mean (SD)^b^	103	11.47 (2.61)	71	13.95 (3.86)	*U* = 1741.5	<**0.001**
Handedness according to EHI Score, Right/Ambidextrous/Left^c^	134	115/3/16	138	124/1/13	–	0.459
Estimated premorbid intelligence (Danish Adult Reading Test (DART)), Mean (SD) [Mean *Z*-score]^d^	122	22.11 (8.51) [−0.59]	139	26.65 (7.63) [0.0]	*t* = −4.54	**<0.001**
Estimated intelligence based on WAIS, Mean *Z*-score^e,f^	69	−1.26	79	0.0	–	–
Estimated intelligence based on WAIS-III, Mean *Z*-score^e,g^	52	0.73	59	0.0	–	–
PANSS, positive, Mean (SD)	134	20.12 (4.36)	–	–	–	–
PANSS, negative, Mean (SD)	134	21.00 (6.69)	–	–	–	–
PANSS, general, Mean (SD)	134	39.20 (9.57)	–	–	–	–
PANSS, total, Mean (SD)	134	80.32 (16.45)	–	–	–	–
DUI, weeks, Mean (SD)^h^	96	113.51 (163.64)	–	–	–	–

Analyses were performed on subjects with available data. Some variables were not available for all cohorts, hence the varying *N*. Significant *p*-values (*p* < 0.05) are in bold. Handedness was determined with The Edinburgh Handedness Inventory (EHI)^58^.

Duration of untreated illness (DUI) was registered and defined as the time from initial decline in functioning estimated as a consequence of unspecific symptoms related to psychosis^59^.

*P-SES* parental socioeconomic status, *EHI* Edinburgh Handedness Inventory score, *PANSS* Positive and Negative Syndrome Scale, *DUI* duration of untreated illness.

^a^Pearson *χ*^2^ test.

^b^Mann−Whitney *U* test.

^c^Fisher’s exact test.

^d^Two-sample *t* test with pooled variance estimates.

^e^A combined score based on the Similarities and Vocabulary subtests from WAIS/WAIS III: Wechsler Adult Intelligence Scale (Wechsler Adult Intelligence Scale®), presented as *Z*-scores standardized from the mean and standard deviation of the healthy control sample.

^f^Only data from cohorts A and B.

^g^Only data from cohort C.

^h^Only data from cohorts B and C.

Diagnoses were ascertained using the Schedule for Clinical Assessment in Neuropsychiatry Version 2 (SCAN)^21^. Included patients met the diagnostic criteria of schizophrenia (*n* = 138) according to the ICD-10 Classification of Mental and Behavioural Disorders. Exclusion criteria were any previous exposure to antipsychotics or methylphenidate. Antidepressant treatment was not allowed within 1 month prior to baseline examinations, and all assessments were carried out before treatment was initiated. At baseline, patients underwent physical and neurological examinations. Recreational substance use was accepted, but patients with current substance dependency were excluded. Symptom severity was assessed with the Positive and Negative Syndrome Scale (PANSS)^22^.

Healthy controls (HCs) (*n* = 151) were recruited from the community in the Capital Region of Copenhagen through online advertisement. Healthy controls were matched to patients on age, gender, and parental socioeconomic status. For HCs, the exclusion criteria were current or previous psychiatric illness, drug abuse, and a family history of psychiatric illness in a first-degree relative.

Based on an individual assessment, patients and HCs were excluded if they had serious physical illness or a history of head injury with unconsciousness for more than 5 min. Obvious pathology on MRI scans resulted in exclusion from the study.

All procedures were approved by the Ethical Committee of Copenhagen and Frederiksberg/The Capital Region (KF 01-078/97 01-012/98) and the Danish National Committee on Biomedical Research Ethics (H-D-2008-088). Permission to retrieve data from registers was granted by the Danish Data Protection Agency (CSU-FCFS-2017-012). All participants provided written informed consent.

### Definitions of treatment response

Treatment response was determined at two time-points: The *short-term treatment response* was a continuous variable and defined as the relative change in PANSS total score from baseline to short-term follow-up, calculated as (PANSS_Follow-up_ − PANSS_Baseline_)/PANSS_Baseline_. Short-term follow-up examinations were conducted after 3 months (cohort A), 6 months (cohort B), and 6 weeks (cohort C).

The *long-term treatment response* was a binary, categorical variable and defined using the criteria presented in Wimberley et al.^23^, which are based on data from the Danish National Prescription Registry, the Danish Psychiatric Central Research Register, and the Danish National Patient Registry. Accordingly, poor long-term responders fulfilled at least one of the following criteria from inclusion to December 12, 2016 based on data from the Danish National Health Service Prescription Database and the Danish Psychiatric Central Research Register linked to participants via their unique personal identification number: (1) *Clozapine prescription*, defined as at least one pharmacy redemption of clozapine; (2) *Eligibility for clozapine*, defined as two nonoverlapping periods of minimum 6 weeks duration treated with different antipsychotics followed by hospital admission; (3) *Polypharmacy*, defined as >90 consecutive days of treatment with at least two different antipsychotics. The definition of poor long-term treatment response overlaps with treatment resistance, but is not identical to the criteria specified by Howes et al.^2^. The average time for assessment of long-term response was 16.9 years (standard deviation (s.d.) = 1.1 years) for cohort A, 10.8 years (s.d. = 1.0 years) for cohort B, and 6.0 years (s.d. = 1.4 years) for cohort C. The overall average was 10.1 years (s.d. = 4.2 years).

### Explanatory variables

#### Cognition

A Danish version of the National Adult Reading Test (DART) was used to estimate premorbid intelligence^24^. Verbal intelligence was estimated using the Vocabulary and Similarities subtests from either WAIS^25^ or WAIS-III^26^, and nonverbal intelligence was estimated using the Block Design and Matrix Reasoning subtests from WAIS-III. Selected tests from the Cambridge Neuropsychological Test Automated Battery (CANTAB) were used to obtain measures of spatial span (SSP), spatial working memory (SWM), spatial planning (Stockings of Cambridge [SOC]), intra-extra dimensional set shifting (IED), sustained attention (Rapid Visual Information Processing [RVP]), and simple reaction and movement times (RTI)^27^. The Brief Assessment of Cognition in Schizophrenia (BACS) was used to assess fluency, working memory, verbal memory, motor skills, processing speed, and planning^28^. Buschke Selective Reminding Test^29^ was used to assess verbal memory, the Symbol Digit Modalities Test^30^ and Trail Making tests A and B^31^ were used to assess processing speed. Wisconsin Card Sorting Test^32^ was used to assess set shifting, and the Speed and Capacity of Language Processing Test^33^ was used to assess speed of verbal processing.

#### Magnetic resonance imaging data

High-resolution T1-weighted structural magnetic resonance images (sMRI) were acquired on three different scanners. In cohort A we used a 1.5 T Siemens Vision scanner with the scanner’s birdcage transmit/receive head coil (Siemens Healthcare, Erlangen, Germany). In cohort B we used a 3.0 T Siemens MAGNETOM trio scanner (Siemens Healthcare) with an eight-channel SENSE head coil (Invivo Corporation, Gainesville, FL), and in cohort C we used a 3.0 T Philips Achieva scanner (Philips Healthcare, Best, The Netherlands) with a SENSE eight-channel head coil (Invivo Corporation).

FreeSurfer Version 5.3.0 was used to process all images as described in Jessen et al.^34^ and in the FreeSurfer documentation^35–37^. Regional measures of cortical thickness, surface area, and mean curvature were identified using the Desikan−Killiany atlas^38^. Subcortical volumes were identified using the anatomical processing pipeline (fsl_anat) (FSL version 5.0.10, FMRIB, Oxford, UK)^39^. Details on scanner settings and image processing are provided in Supplementary Text S1.1.

#### Electrophysiology data

All participants were examined using parts of the Copenhagen Psychophysiology Test Battery (CPTB). The CPTB consists of the prepulse inhibition (PPI), P50 suppression, mismatch negativity (MMN), and selective attention (SA) paradigms. Methods have previously been described in detail^40–44^ (see also Supplementary Text S1.2 and Supplementary Table S1).

#### Register data

Register data on all participants were obtained from The Danish Medical Birth Register hosted at the Danish Health Data Authority by data linkage using the unique personal identification number as key. We used data on maternal and paternal age at birth, gestational age in weeks, birth length and weight, and Apgar scores after 1 and 5 min.

#### Covariates

In all analyses, the conventional covariates: sex, age, cohort, and handedness were used. The cohort covariate primarily accounts for differences in the time of assessment, differences in antipsychotic compound, and different MRI scanners.

### Missing data

In this study we have pooled data from three comparable cohorts. The pooled sample had both block-wise and randomly missing data.

**Fig. 1:**
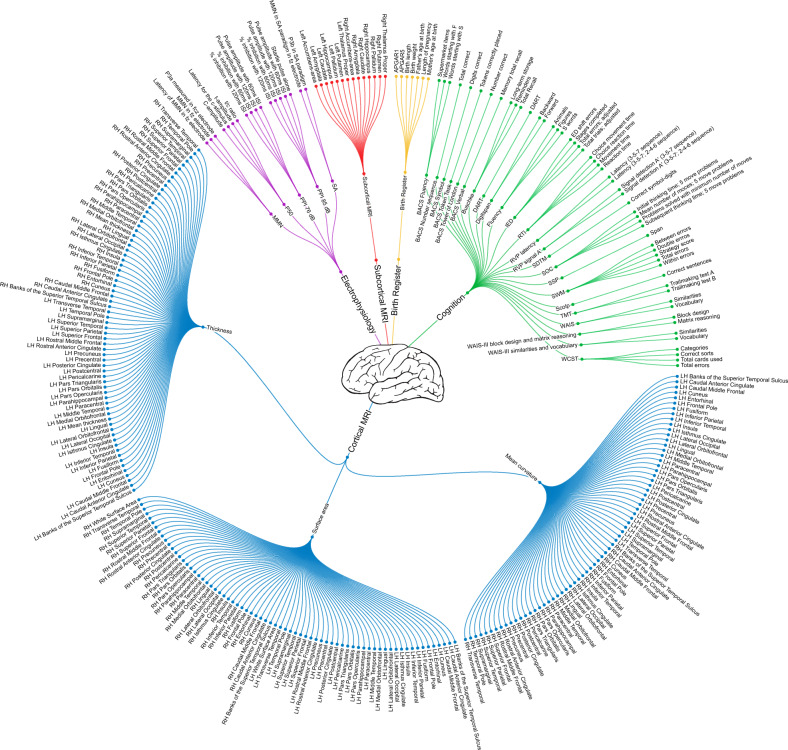
Radial dendrogram depicting our data model. Modalities were divided into submodalities, each with a set of features. The nodes closest to the center (depicted as a brain) represent the modalities. Distal to these are the submodalities, and along the circumference are the leaves representing the features (i.e. the variables). MRI magnetic resonance imaging, LH left hemisphere, RH right hemisphere, MMN mismatch negativity, PPI prepulse inhibition, SA selective attention, ISI inter-stimulus-interval, APGAR Appearance Pulse Grimace Activity Respiration, BACS Brief Assessment of Cognition in Schizophrenia, Buschke Buschke Selective Reminding Test, DART Danish version of the National Adult Reading Test, IED Intra-Extra Dimensional Set Shifting, RTI reaction time, RVP Rapid Visual Information Processing, SDTM Symbol Digit Modalities Test, SOC Stockings of Cambridge, SSP Spatial Span, SWM Spatial Working Memory, SCOLP Speed and Capacity of Language Processing, TMT Trail Making Test, WAIS Wechsler Adult Intelligence Scale, WCST Wisconsin Card Sorting Test. For a description of electrophysiology features, see Supplementary Table S1.

To handle block-wise missing data, we divided each modality into submodalities. Subsequently, we integrated the predictions of each submodality, i.e. late integration. An overview of submodalities and their features is provided in Fig. 1. We tested two different integration schemes on the simulated data (for details see Supplementary Text S1.3).

Randomly missing data were handled by applying imputation^45^. To reduce bias in our results, we tested two different imputation methods on the simulated data: median imputation and probabilistic principal component analysis (PPCA) imputation^46,47^.

Details on handling of missing data can be found in Supplementary Text S1.3.

### Analysis strategy

#### Simulated data

In order to minimize bias in algorithm selection and parameter settings, we produced simulated datasets. The simulated data facilitated unbiased choices in the subsequent learning process (e.g. late data integration and imputation). The simulated data resembled the actual data with respect to dimensionality, multimodality, and pattern of missing data. Tunable noise levels allowed us to evaluate performance and robustness across different signal-to-noise ratios (SNRs). The simulated data were matched to the real data by creating a “simulated patient” for each true patient.

We simulated data by sampling from a *latent variable model*^48^. The underlying assumption of our model was that each subject has a latent variable, which reflects his/her capability of responding to treatment. Based on two common hypotheses of the underlying nature of treatment response of schizophrenia patients, we imposed two restrictions on the one-dimensional latent variable^2,17,49^. This resulted in two datasets, denoted cluster data and spectrum data, respectively (see Supplementary Text S1.4).

The pattern of missing data extracted from the real data was applied to the simulated data. In total, 180 simulated datasets were generated by varying the SNR from −20 to 20 dB in steps of 5 dB, using two data types, and by initiating the data generation process using ten different random seeds.

Details on the generation of simulated data can be found in Supplementary Text S1.4.

#### Machine-learning framework

**Fig. 2:**
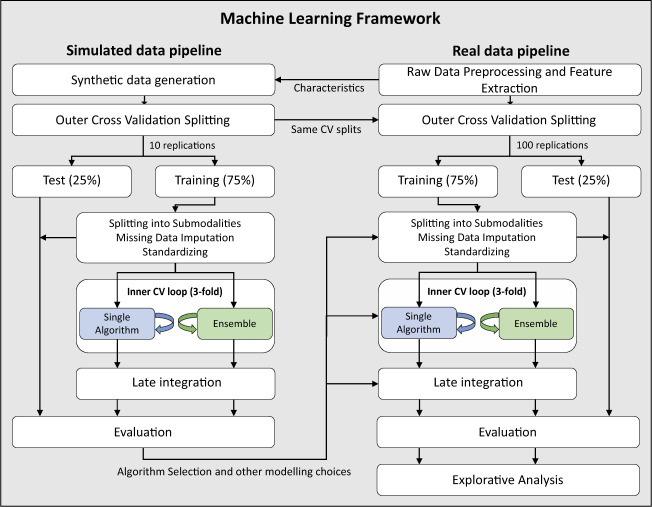
Overall machine-learning framework using both simulated and real data. In the outer CV loop, the data were randomly split, leaving out 25% of the subjects for testing with 10 replications for the simulated data and 100 replications for the real data. The subjects were stratified with respect to cohort (short-term treatment response) or outcome (long-term treatment response and diagnostic classification). Values missing at random were imputed using estimates derived from the training set. The training and test sets were standardized by the mean and standard deviation derived from the training set. Both training and test data were split into submodalities. We used the following conventional covariates: sex, age, cohort, and handedness. In the inner CV loop, the training data were further split into a training and test set using threefold CV. Threefold CV was selected as a tradeoff between limited sample size and computation time. Algorithm parameters and ensembles were optimized in the inner CV loop with two different approaches (see text). The best performing model was applied to the outer CV loop test set and the prediction of each submodality was combined in a late integration scheme to provide the prediction. The analysis of the real data followed the same framework as the simulated data, except that only the best, median and poorest performing algorithms, parameter settings, and methods learned from the simulated data were applied on the real data. CV cross-validation.

The overall ML framework is outlined in Fig. 2.

In order to ascertain the robustness of the ML framework, we applied it using two independent approaches denoted “single algorithm approach” and “ensemble approach,” respectively (code available from https://lab.compute.dtu.dk/cogsys_lundbeck_cnsr/schizophrenia_treatment_resistance/).

The *single algorithm approach* was implemented in Matlab Release 2018a (The MathWorks, Inc., Natick, Massachusetts, USA). For prediction of the continuous short-term treatment response, we tested nine Matlab built-in regression algorithms with different settings resulting in 32 configurations. The regression algorithms tested were *linear regression* algorithms, *support vector machines* (SVMs) with different kernels, *Gaussian Processes*, *regression trees*, *generalized linear models*, *ensemble regression* algorithms, and *random forest*. For prediction of the binary long-term treatment response and diagnostic classification, we tested eight Matlab built-in classification algorithms with different settings, resulting in 21 configurations. The classification algorithms tested were *logistic regression*, *Naïve Bayes*, *random forest*, *decision trees*, *ensemble of trees*, *SVMs* with different kernels, and *k-nearest neighbor*. In some configurations, one or more of the parameters were optimized with Bayesian optimization in the inner cross-validation (CV) loop, while in others the default settings were used. To validate our methodological framework, we identified the best, the median, and the poorest performing algorithms in the single algorithm approach, to investigate if the performance ranking of the algorithms on the simulated data were kept in the real data.

The *ensemble approach* was implemented in Python (version 2.7.15+) using auto-sklearn (version 0.4.1)^50^. Auto-sklearn is an open-source ML framework, which automatically performs ML algorithm selection, hyperparameter tuning and builds an ensemble of the selected algorithms. Each algorithm in the ensemble, as well as the ensemble itself, was fine-tuned automatically using Bayesian optimization. The impact of two main parameters in auto-sklearn was tested on simulated data, specifically the time limit in seconds to search for appropriate algorithms and the optimal ensemble (denoted training time), and the maximum number of algorithms included in the final ensemble (denoted maximum ensemble size), respectively. We tested the performance in a grid search using 20, 60, and 180 s, as well as maximum ensemble sizes of 1, 4, and 40, to find the optimal combination of training time and maximum ensemble size. Validation of the ensemble approach consisted of testing if the combination of short training time and small maximum ensemble size worsened our results and likewise, if the combination of long training time and large maximum ensemble size improved our results when applied to the real data.

### Model performance measures

The performance of the classification algorithms (for diagnostic classification and estimation of long-term treatment response) was calculated as the balanced accuracy (BACC). Balanced accuracy is useful when the classes are of unequal sizes. For random classification the BACC will give a score of 0.5, whereas a BACC of 1 means perfect classification.

The performance of the regression algorithms (i.e. estimation of short-term treatment response) was assessed by normalized mean square error (NMSE). An NMSE of 0 means perfect prediction, whereas an NMSE of 1 equals chance level. Details on model performance measures can be found in Supplementary Text S1.5.

### Statistical analyses

Demographic and clinical data were analyzed using the Statistical Package for the Social Sciences software (version 25, SPSS Inc., USA). The distribution of continuous data was tested for normality with the Shapiro−Wilk test and by visual inspection of histograms. Depending on the distribution and type of the data, the group differences were tested using a two-sample *t* test, the Mann−Whitney *U* test, Pearson’s *χ*^2^ test, or Fisher’s exact test (Table 1).

## Results

### Group differences

**Fig. 3:**
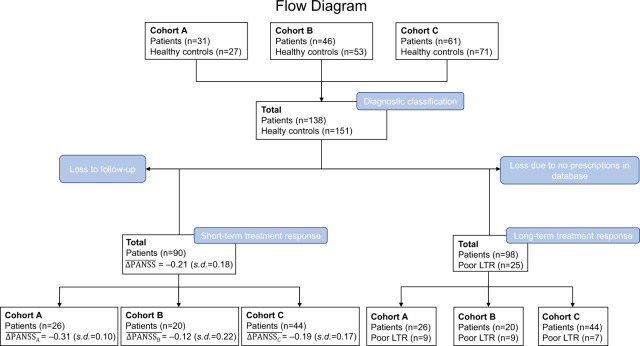
Flow diagram of subject inclusion into diagnostic classification and prediction of short- and long-term treatment response. $\overline {{\Delta}{\mathrm{PANSS}}}$ mean relative change in Positive And Negative Syndrome Score, s.d. standard deviation, LTR long-term treatment response.

For a flow diagram of the study, refer to Fig. 3.

In total, 51.1% of the subjects had a relative decrease in PANSS score of minimum 20% (for details see Fig. 3).

In the definition of long-term treatment response, 25 patients were classified as poor long-term responders based on the aforementioned criteria: clozapine prescriptions (*n* = 4), eligible for clozapine (*n* = 5), and polypharmacy (*n* = 16).

Patients and controls differed in estimated premorbid intelligence and years of education (Table 1). No other group differences were identified.

### Simulated data results

The results of the simulated data are shown in Supplementary Fig. S1. As expected, the higher the SNR, the higher prediction accuracy. In the extreme cases with either very high or very low SNR, all algorithms performed equally well or poorly. However, in the low SNR range, it was possible to differentiate between the algorithms and parameter settings and select the best performing combination (i.e. low error and stable performance across the SNR interval) for the given problem. For each combination of data type (i.e. cluster or spectrum), seed, and SNR in the range [−20, 0], we found the best performing algorithm in the single algorithm approach and the best combination of training time and maximum ensemble size in the ensemble approach. The models that performed best on average was subsequently applied to the real data.

### Single algorithm approach

#### Diagnostic classification

The best performing algorithm across the simulated datasets when classifying patients from HCs was the *ensemble of trees* algorithm with Bayesian optimization of the hyperparameters (*fitcensemble* in Matlab). Applying this algorithm on real data yielded a BACC of 64.2% (confidence interval (CI): [51.7, 76.7]).

To validate our methodological framework, we tested if the ranking of the algorithms from the simulated data was kept in the real data. We did this by identifying the best, the median, and the poorest performing algorithms, namely the *ensemble of trees* algorithm with Bayesian optimization (top performance), a *logistic regression* algorithm (middle performance), and an *SVM* with radial basis function kernel function (poorest performance). Their performances and CIs on the real data are listed in Table 2. The ranking of the algorithms was kept in the real data; however, there was no significant difference between the *ensemble of trees* algorithm with optimization and the *logistic regression* algorithm (*p* = 0.52).

**Table 2:** Performance and confidence intervals of the selected algorithms when predicting the three different problems.

Single algorithm approach		
Diagnostic classification	BACC (%)	95% confidence interval
Best performance: *Ensemble of trees with Bayesian optimization*	**64.2**	**[51.7, 76.7]**
Medium performance: *Logistic regression*	**63.8**	**[50.7, 77.0]**
Worst performance: *SVM with radial basis function kernel*	50.4	[44.0, 56.8]
Long-term treatment response (classification)		
Best performance: *Logistic regression for high-dimensional data*	50.3	[39.4, 61.2]
Medium performance: *Random forest*	49.7	[44.7, 54.6]
Worst performance: *Linear SVM*	50.0	[50.0, 50.0]
Short-term treatment response (regression)	NMSE	95% confidence interval
Best performance: *SVM with L1 regularization*	0.96	[0.43, 1.49]
Medium performance: *Linear regression with L1 regularization*	0.96	[0.42, 1.51]
Worst performance: *SVM with polynomial kernel*	14.86	[0, 35.09]

Ensemble approach		
Diagnostic classification	BACC (%)	95% confidence interval
Chosen settings based on simulated data results: maximum ensemble size = 4, training time = 180 s	**63.8**	**[50.8, 76.7]**
Small maximum ensemble size (=1) and short training time (=20 s)	56.8	[48.1, 65.4]
Large maximum ensemble size (=40) and long training time (=180 s)	**63.6**	**[50.7, 76.5]**
Long-term treatment response (classification)		
Chosen settings based on simulated data results: maximum ensemble size = 1, training time = 60 s	50.0	[50.0, 50.0]
Small maximum ensemble size (=1) and short training time (=20 s)	50.0	[50.0, 50.0]
Large maximum ensemble size (=40) and long training time (=180 s)	50.0	[50.0, 50.0]
Short-term treatment response (regression)	NMSE	95% confidence interval
Chosen settings based on simulated data results: maximum ensemble size = 40, training time = 180 s	1.04	[1.04, 1.04]
Small maximum ensemble size (=1) and short training time (=20 s)	1.06	[1.06, 1.06]

Balanced accuracy and NMSE are averaged across 100 cross-validation splits. Values in bold are significant on a 95% confidence level. BACC, balanced accuracy.

*NMSE* normalized mean squared error, *SVM* support vector machine.

Post-hoc analyses showed that the classification was primarily driven by the cognitive data (see Supplementary Table S2). Classifying patients from controls based on cognition only yielded a BACC of 67.8% (CI: [54.7, 81.0]), which was significantly higher than using all modalities (*p* < 0.01, Supplementary Table S2).

#### Long-term treatment response

For prediction of the long-term treatment response, we selected a *logistic regression* algorithm developed for high-dimensional data (*fitclinear* in Matlab). The average BACC across 100 CV splits was 50.30%, which is indistinguishable from random guessing.

#### Short-term treatment response

When predicting the short-term treatment response, we selected an *SVM* with L1 regularization, which yielded a nonsignificant prediction (NMSE = 0.96).

### Ensemble approach

#### Diagnostic classification

When classifying patients from HCs, the best performing combination of parameters across the simulated datasets was a maximum ensemble size of 4 and a training time of 180 s. Applying this combination on the real data yielded a significant BACC of 63.8% (CI: [50.8, 76.7]). Decreasing the training time and maximum ensemble size worsened model performance on the real data and increasing the maximum ensemble size to 40 did not improve the result (BACC = 63.6%) either. Post-hoc analyses showed that correct classification was driven by the cognitive data with a BACC of 63.8% (CI: [52.0, 75.5], see Supplementary Table S3).

#### Long-term treatment response

For prediction of the long-term treatment response, the best performing combination of parameters on the simulated data was a maximum ensemble size of 1 and a training time of 60 s. The average balanced accuracy across 100 CV splits was 50.0% (Supplementary Table S3). Neither decreasing nor increasing the training time and maximum ensemble size changed the results when applied to the real data.

#### Short-term treatment response

When predicting the short-term treatment response, the best performing combination of parameters on the simulated data was a maximum ensemble size of 40 and a training time of 180 s, which was the maximum training time and maximum ensemble size tested. This combination yielded a nonsignificant prediction (NMSE = 1.04). Reducing the training time and maximum ensemble size insignificantly increased the NMSE when applied to the real data.

## Discussion

Here, we have presented a novel and robust framework for applying ML to multimodal data, while accounting for missing data and reducing bias in model selection and fine-tuning. Our single algorithm and ensemble approaches produced consistent results, and both approaches were able to significantly classify schizophrenia patients from HCs above chance level. However, neither approach predicted the treatment response.

Additional calculations performed on unimodal data revealed cognition to be the primary driver of the significant results, and cognition alone was, in the single algorithm approach, superior to using multimodal data (Supplementary Tables S2, S3). The strong cognitive signal is in line with our recent findings^16^, which were reported on data partly overlapping with the data in the current study (cohort C). Interestingly, Doan et al. found that a *random forest* classifier performed better when combining cognition data and MRI data from a linked independent component analysis rather than using cognition data alone^15^. Even so, no direct comparison can be made to our study, since the schizophrenia patients included in the study by Doan et al. were not antipsychotic-naïve, which may have enhanced the MRI signal in their data.

We strived to be unprejudiced and “agnostic” in our selection of input data by including comprehensive data from several modalities. This was done to minimize the risk of leaving out data that could improve model performance, but also meant that we risked reducing the SNR by adding data that would primarily introduce noise. It could be speculated that use of “domain knowledge,” i.e. to include only submodalities and features which have been clearly implicated in schizophrenia in the literature, may have provided different results.

In the current study, schizophrenia patients and HCs differed significantly in completed years of education and estimated premorbid intelligence, but not in parental socioeconomic status. This was expected since illness onset impacts educational attainment, and a large majority of schizophrenia patients function at a lower cognitive level than that predicted by their parental socioeconomic status^51,52^.

Theoretically, the selection of only one algorithm in the single algorithm approach could be problematic when the dimensionality of the submodalities vary, because the same algorithm may not be the optimal choice for all submodalities. In contrast, the ensemble approach could optimally account for each submodality separately, and one would expect the more flexible approach to perform better on a highly complex multimodal dataset, though possibly at the expense of interpretability. Regardless of the differences between the two approaches, their overall predictions on our dataset were very similar.

Observational multimodal studies are often limited by the number of participants. In turn, a limited number of observations leaves less independent clinical data to test the ML models on. To overcome this limitation, CV can be performed. Using randomized splits of the data, CV stabilizes algorithm performance. However, to reduce biases in the CV average, this can only be done once, i.e. multiple algorithms cannot be tested without biasing the result.

The methodological framework presented herein, incorporating simulated data, two parallel ML approaches, and nested CV, helps to reduce bias in algorithm and parameter selection and to obtain reliable results with modest sample sizes when independent replication samples are not available.

The application of simulated data in our framework also provided an unbiased way of evaluating algorithm performance before the test phase. Furthermore, the performance ranking of the models on simulated data was robustly translated to the real data. Still, we cannot know the actual performance of every model on real data without having tested it but doing so would increase the risk of type 1 errors. Likewise, we only applied the top performing model from the multimodal analyses on simulated data when conducting post-hoc analyses on unimodal data (see Supplementary Tables S2, S3).

Due to block-wise missing data, modeling submodalities rather than complete modalities allowed us to retain a larger number of subjects in the analyses without performing massive imputation. The late integration approach also facilitated clinical interpretation of the results. A drawback of late integration is that correlations between submodalities are not considered. However, intra-submodality correlations are still preserved.

We handled randomly missing data by using imputation (for details see Supplementary Text S1.3). Imputation may introduce noise to a dataset^53^ and could be part of the reason our framework was not able to predict treatment response.

We used a one-dimensional latent variable to reflect the capability of treatment response of each subject, which may have been too restrictive to effectively model the disease. Though we applied two different models of the latent variable, more complex data could have been generated. However, the choices were made for simplification, while still capturing characteristics of the real data.

In the SNR interval [−20; 0] we found a discernible span in algorithm performance in the single algorithm approach. The true SNR could lie outside of this interval, in which case our framework would not provide any meaningful guidance regarding choice of algorithm.

Included patients were moderately ill at baseline (average total PANSS of 80.3). As such, this study, like all studies of voluntary participants suffering from schizophrenia, may be limited by selection bias, since the most severely psychotic and agitated patients will not be able to provide informed consent, let alone undergo e.g. MRI.

Although the relative change in total PANSS score is commonly considered a relevant measure of treatment response, other more specific symptom domains might have been informative. However, to limit the number of tests, we restricted our analyses to this measure.

About 25% of the subjects originally included in the cohorts had not redeemed any prescriptions at the time of evaluation of long-term treatment response. Possible explanations for this include patients that have gone into remission or have discontinued their medication. Moreover, patients that are hospitalized or attending specialized outpatient clinics (OPUS clinics^54^) do not have their medication registered in the Danish prescription database.

We could not use treatment resistance as outcome, since we did not have data regarding e.g. adherence^2^. Using the “Wimberley criteria” in the definition of poor long-term treatment response, the poor responders in our sample primarily consisted of patients on polypharmacy. The low percentage of clozapine eligible patients in our sample could indicate that some aspects of psychosis are less represented as compared to a general clinical population. Still, part of the patients without prescriptions could be undiscovered poor long-term responders if, for instance, they discontinued their medication due to psychotic symptoms. We did not have information as to what degree patients responded to antipsychotic treatment after the initial trial intervention period. Some patients may, despite symptom improvement, have changed medication due to side effects.

In some cases, patients develop treatment resistance after years of previously effective antipsychotic treatment. This could also be the case with our definition of long-term treatment response. A proportion of the patients will most likely change status to poor long-term responders at some point after the inclusion date for the present study. This entails an implicit cohort bias since patients included in the first cohorts will have had longer time to become poor long-term responders than those recruited later. Even so, all patients had been ill for >2 years prior to inclusion in this study, compared to the minimum 12 weeks of illness that are required to meet the TRRIP criteria for treatment resistance^2^. We also sought to mitigate cohort bias by including cohort as a covariate in our analyses.

Since all our input data were collected cross-sectionally at baseline, we could not account for any changes in the neuropsychiatric measurements. Dynamic changes in e.g. brain structure in first-episode schizophrenia patients may compromise the utility of cross-sectional neuroimaging data to function as a biomarker and measurement trajectories may be better suited for this purpose^55^. However, by applying cross-sectional neuropsychiatric data from multiple modalities, we aimed to leverage this potential source of variability.

Using sparse canonical correlation analysis, Doucet et al.^56^ found correlations between baseline functional connectivity in several brain networks and clinical response after antipsychotic medication. They did not find any significant associations between clinical outcome and cortical thickness, subcortical volumes, or a combination of structural and resting-state functional MRI measurements. This suggests that our multimodal setup might have benefitted from incorporating functional MRI data. However, the participants in the study by Doucet et al. were not antipsychotic-naïve and included patients past their first psychotic episode; hence part of the signal may be attributable to the more chronic patient sample.

In order to maximize sample size, we combined data from three different cohorts. In the case of the MRI modalities, this meant pooling data from scanners of variable field strengths and from different manufacturers. Cohorts also varied with regards to which antipsychotic compound patients were treated with, exact dosage, and the length of the treatment period before short-term follow-up. These variations may in turn have increased sample heterogeneity and “diluted” the signal necessary for the ML algorithms to effectively solve the three problems.

In future work, there are several other mechanisms that could be tested. These include alternative late integration schemes, as well as other imputation types, such as multiple imputation and imputation with reject option^17,57^.

In summary, our rigorous modeling framework involving simulated data and two parallel ML approaches significantly discriminated patients from controls. However, our extensive neuropsychiatric data from antipsychotic-naïve patients were not predictive of treatment response. Validation of the framework showed that the ranking of the algorithms and parameter settings in the simulated data was maintained in the real data. In conclusion, this novel framework holds promise as an important step to minimize bias and obtain reliable results with modest sample sizes when independent replication samples are not available.

## Supplementary information

Supplemental Material

Table S1

Figure S1

Table S2

Table S3
