# Supplementary material for: A machine-learning framework for robust and reliable prediction of short- and long-term treatment response in initially antipsychotic-naïve schizophrenia patients based on multimodal neuropsychiatric data
Source: Transl Psychiatry. 2020 Aug 10;10:276. doi: 10.1038/s41398-020-00962-8 (PMC7417553; doi:10.1038/s41398-020-00962-8)
Supplement: Supplementary file 1 — Supplemental Material [file 41398_2020_962_MOESM1_ESM.docx]

A machine learning framework for robust and reliable prediction of short- and long-term treatment response in initially antipsychotic-naïve schizophrenia patients based on multimodal neuropsychiatric data.

Authors

Karen S. Ambrosen^a,*^, Martin W. Skjerbæk^a,*^, Jonathan Foldager^b,*^, Martin C. Axelsen^a,b^, Nikolaj Bak^c^, Lars Arvastson^c^, Søren R. Christensen^c^, Louise B. Johansen^a,d^, Jayachandra M. Raghava^a,e^, Bob Oranje^a,g^, Egill Rostrup^a^, Mette Ø. Nielsen^a,f^, Merete Osler^h,i^, Birgitte Fagerlund^a,j^, Christos Pantelis^a,k^, Bruce J. Kinon^l^, Birte Y. Glenthøj^a,f^, Lars K. Hansen^b^ and Bjørn H. Ebdrup^a,f^

# S1. Supplementary Text

## S1.1. Image acquisition and processing

Details on scanner settings for the 3 cohorts:

*Cohort A*: T1-weighted sagittal MPRAGE images were obtained with echo time (TE) 4 ms, repetition time (TR) 9.7 ms, flip angle 12°, field of view (FOV) 250 mm, matrix 256 × 256, 0.98 × 0.98 × 1 mm^3^ voxels, 170 slices.

*Cohort B*: T1-weighted MPRAGE images were acquired with TE 3.93 ms, TR 1540 ms, flip angle 9°, FOV 256 mm, matrix 256 × 256, 1 mm isotropic voxels, 192 slices.

*Cohort C*: T1-weighted FFE images were acquired with TE 4.6 ms, TR 10 ms, flip angle 8°, FOV 240 mm, matrix 304 x 299, acquired voxel size 0.79 x 0.80 x 0.80 mm3 and reconstructed voxel size 0.75 x 0.75 x 0.80 mm3, 200 slices.

The cohorts were part of longitudinal studies, and the images were processed using the longitudinal processing pipeline in FreeSurfer. Only segmentations of the baseline images were used in the present study, and we applied a 3T specific option for Talairach alignment (i.e. Schwartz atlas) (FreeSurfer Previous Release Notes, 2013) for the images acquired on a 3T scanner (*Cohorts B & C*).

Segmentation of subcortical volumes were conducted in several steps, including neck removal, bias-field correction^1^, brain extraction^2^, tissue type segmentation^1^, and FIRST^3^. All segmentations were visually inspected by a blinded rater to ensure sufficient quality of the data included in the analyses.

## S1.2. Electrophysiology

Auditory stimuli were presented binaurally through stereo insert earphones (Eartone ABR, 1996–2008 Interacoustics A/S, Assens, Denmark; and C and H Distributors Inc, Milwaukee, WI, USA) via a computer running ‘Presentation’ software (Neurobehavioral Systems, Albany, NY, USA) (soundcard: Creative Sound Blaster 5.1, 2008 Creative Technology, Singapore).

Electroencephalography (EEG) was recorded using a cap with 64 active electrodes (BioSemi, Amsterdam, The Netherlands). For PPI, electromyography (EMG) activity in the right orbicularis oculi muscle was recorded to measure the eye-blink component of the acoustic startle response. Processing of the data was performed with BESA software (version 5.2.4, MEGIS Software, Gräfelfing, Germany). In the case of the PPI paradigm, a continuous 70 dB of white noise was played in the background. Prepulse volume was either 76 dB or 85 dB and the time between prepulse and pulse (ISI) was either ms or 120 ms.

Electrophysiology was divided into 5 submodalities each with a set of features ranging from 2-5 (Table S2).

## S1.3 Missing Data

In this study we have pooled data from three comparable cohorts. The pooled sample had both block-wise and randomly missing data.

Block-wise missing data occurs, when a variable is systematically missing in a cohort, e.g. due to differences in the study design or because of concurrent scientific development within a modality. Data missing at random occurs if single values are missing, e.g. if technical errors compromise data quality.

To handle block-wise missing data we divided each modality into submodalities. This enabled each submodality to be modelled independently of the other submodalities. Subjects with block-wise missing data were excluded from that specific analysis. Subsequently, we integrated the predictions of each submodality, i.e. late integration. An overview of submodalities and their features is provided in Figure 1.

We tested two late integration schemes: majority voting and probabilistic voting. In majority voting, the final prediction reflects the average prediction and the majority of predictions across submodalities for the regression and classification problems, respectively. In probabilistic voting, in addition to the prediction, the classification score of the algorithms is also considered. Hence, submodality predictions with high classification scores are given higher weights. We used the late integration scheme with probabilistic voting from Axelsen et al.^2^.

Randomly missing data was handled by applying imputation^3^. To reduce bias in our results, we tested two different imputation methods on the simulated data: median imputation and probabilistic principal component analysis (PPCA) imputation^4,5^.

Median and PPCA imputation showed similar performance on the simulated data (results not shown), hence we selected median imputation based on computational efficiency. We also tested probabilistic voting against majority voting. Since probabilistic voting generally provided less stable results (results not shown), we applied majority voting on the real data.

## S1.4 Generation of Simulated Data

We simulated data by sampling from a *latent variable model*^6^. The assumption is that each subject has a one-dimensional latent variable, $z$, that does not change. The latent variable reflects his/her capability of responding to treatment. The latent variable $z$ cannot be observed directly but is projected differently onto each feature in the data set $X$. $X$ has the dimensions $N\times F$, where $N$ is the number of subjects and $F$ is the number of features. This can be written as that does not change. The latent variable reflects his/her capability of responding to treatment. The latent variable $z$ cannot be observed directly but is projected differently onto each feature in the data set $X$. $X$ has the dimensions $N\times F$, where $N$ is the number of subjects and $F$ is the number of features. This can be written as

$$x_{n,f}=z_{n}w_{f}+\epsilon_{n,f},$$

Where $x_{n,f}$ is the datapoint for the $n$’th subject and the $f$’th feature, $z_{n}$ is the latent variable for subject $n$, $w_{f}$ is the projection weight of feature $f$, and $\epsilon_{n,f}$ is added noise, where $\epsilon\mathcal{\sim N}\left( 0,\sigma^{2} \right)$. The relation between $\sigma^{2}$ and SNR is given by

$$\sigma^{2}=\frac{1}{N}\sum_{n=1}^{N} \left( z_{n}w_{f}-\bar{zw_{f}} \right)^{2}{10}^{-SNR_{dB}/10},$$

where $SNR_{dB}$ is the SNR in decibel and $\bar{\left( \cdot\right)}$ is the mean value. Based on two different models of the underlying nature of the outcome of schizophrenia we imposed two restrictions on $z$. This procedure resulted in two differently simulated data sets, denoted cluster data and spectrum data, respectively. The cluster data contained two separated classes (clusters). Conversely, the spectrum separated the two classes on a threshold $\delta$ on $z$. The continuous outcome variable, $y$, was generated from $z$ with a projection coefficient $w_{y}$ as $y_{n}=z_{n}w_{y}$. In the classification tasks, we applied the same threshold $\delta$ on $z$ as before, resulting in a binary outcome variable. The components included in the data simulation can be summarized as

|  | Component | Dimensions | Density Function |
| --- | --- | --- | --- |
|  | $\boldsymbol{X}$ | $N\times F$ | $x_{n,f}\mathcal{\sim N}\left( z_{n}w_{f},\sigma^{2} \right)$ |
|  | $\boldsymbol{W}$ | $1\times F$ | $w_{f}\sim\mathcal{N}\left( 0,1 \right)$ |
|  | $\epsilon$ | $N\times F$ | $\epsilon_{n,f}\sim\mathcal{N}\left( 0,\sigma^{2} \right)$ |
| Short-term treatment response | $\boldsymbol{y}$ | $N\times1$ | $y_{n}\sim\mathcal{N}\left( z_{n}w_{y},1^{2} \right)$ |
| Long-term treatment response | $\boldsymbol{y}$ | $N\times1$ | $y_{n}\sim\left\{ \begin{matrix} 1, & \text{if} z_{n}>\delta\\ 0, & \text{if} z_{n}<\delta\end{matrix} \right.$ |
| Spectrum | $\boldsymbol{z}$ | $N\times1$ | $f\left( z \right)\sim\left\{ \begin{matrix} 1, & if 0<z<1 \\ 0, & otherwise \end{matrix} \right.$ |
| Cluster | $\boldsymbol{z}$ | $N\times1$ | $f\left( z \right)\sim\left\{ \begin{matrix} \alpha, & if 0<z<a \\ 1-\alpha, & if b<z<1 \\ 0, & otherwise \end{matrix} \right.$ |

To resemble the structure of the real data, the pattern of missing data extracted from the real data was applied to the simulated data. In total, 180 simulated data sets were generated by varying the SNR from -20 dB to 20 dB in steps of 5 dB, using two data types, and by initiating the data generation process using 10 different random seeds. This strategy was chosen since we do not know the true SNR.

## S1.5. Model Performance Measures

The performance of the classification algorithms (for diagnostic classification and estimation of long-term treatment response) was indicated by balanced accuracy (BACC). BACC is useful when the classes are of unequal sizes. For random classification the BACC will give a score of 0.5, whereas a BACC of 1 means perfect classification. The BACC for each CV split, c, and class, k, was determined by the average accuracy across classes

$$BACC_{c} = \frac{1}{K}\sum_{k} ACC_{c_{k}},$$

where
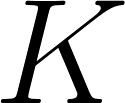
 is the number of classes. The class can be either patient (k=0) or HC (k=1). The accuracy (ACC) for each CV split and class was determined by

$$\mathrm{AC}C_{c_{k}}=\frac{1}{N_{c_{k}}}\sum_{x:y_{\mathrm{true},c}=k} \mathcal{I}\left( y_{\mathrm{true},c}\left( x \right)=y_{\mathrm{pred},c}\left( x \right) \right),$$

where $\mathcal{I}$ is the indicator function, which equals 1 if the prediction is correct and 0 otherwise, and $N_{c_{k}}$ is the number of subjects belonging to class k in the given CV split, c. $BACC_{c}$ was averaged across CV splits to find the performance (BACC) of the model. The confidence interval across CV splits was calculated as

$$CI_{BACC}=BACC\pm z\cdot\sqrt{\frac{1}{C}\sum_{c} SEM_{BACC,c}^{2}}$$

where $z=1.96$ for a 95% confidence level and the standard error of the mean ($SEM_{BACC}$) for each CV split was calculated as

$$SEM_{BACC,c}=\sqrt{\frac{1}{K}\sum_{k} \left( \frac{1}{N_{c_{k}}}ACC_{c_{k}}\left( 1-ACC_{c_{k}} \right) \right)}.$$

The performance of the regression algorithms (i.e. estimation of short-term treatment response) was assessed by normalized mean square error (NMSE). A NMSE of 0 means perfect prediction, whereas a NMSE of 1 equals chance level. NMSE was calculated for each CV split, c, as

$$\mathrm{NMS}E_{c}=\frac{\frac{1}{N_{c}}\sum\left( y_{pred,c}-y_{true,c} \right)^{2}}{\mathrm{var}\left( y_{true,c} \right)},$$

Where $N_{c}$ is the number of subjects in the given CV split, $y_{pred,c}$ and $y_{true,c}$ are the predicted and the true values of the subjects in the given CV split, and $\mathrm{var}\left( \cdot\right)$ is the variance. $NMSE_{c}$ was averaged across CV splits to find the performance of the model. The confidence interval across CV splits was calculated as

$$CI_{NMSE}=NMSE\pm z\cdot\sqrt{\frac{1}{C}\sum_{c} SEM_{NMSE,c}^{2}}$$

Where the standard error of the mean ($SEM_{NMSE}$) for each CV split was calculated as

$$\mathrm{SE}M_{NMSE,c}=\frac{1}{\sqrt{N_{c}}}\sqrt{\frac{1}{N_{c}}\sum\left( \frac{\left( y_{pred,c}-y_{true,c} \right)^{2}}{var\left( y_{true,c} \right)}-NMSE_{c} \right)^{2}}.$$

# Literature

1. Zhang, Y., Brady, M. & Smith, S. Segmentation of brain MR images through a hidden Markov random field model and the expectation-maximization algorithm. *IEEE Trans. Med. Imaging* **20**, 45–57 (2001).

2. Axelsen, M. C., Bak, N. & Hansen, L. K. Testing Multimodal Integration Hypotheses with Application to Schizophrenia Data. in *2015 International Workshop on Pattern Recognition in NeuroImaging* 37–40 (2015). doi:10.1109/PRNI.2015.20.

3. Donders, A. R. T., van der Heijden, G. J. M. G., Stijnen, T. & Moons, K. G. M. Review: A gentle introduction to imputation of missing values. *J. Clin. Epidemiol.* **59**, 1087–1091 (2006).

4. Tipping, M. E. & Bishop, C. M. Mixtures of Probabilistic Principal Component Analyzers. *Neural Comput.* **11**, 443–482 (1999).

5. Hansen, L. K. *et al.* Generalizable Patterns in Neuroimaging: How Many Principal Components? *NeuroImage* **9**, 534–544 (1999).

6. Everitt, B. S. *An introduction to latent variable models*. (Springer Science & Business Media, 2013).

Figure and Table Legends

Table S1: Description of features that were extracted from the modality Electrophysiology. MMN, Mismatch

Negativity; PPI, Prepulse Inhibition; SA, Selective Attention.

Figure S1: Simulated data results from the single algorithm approach (a) and the ensemble approach (b). Each column corresponds to one of the three problems. Each line corresponds to an algorithm or a parameter combination averaged across seeds, cross-validation splits, and data types. The highlighted lines are the best (green), the median (blue), and the worst (red) performing algorithm/setting which are applied to the real data. NMSE, normalized mean squared error; SNR, signal-to-noise ratio; BACC, balanced accuracy

Table S2: Performance and confidence intervals of the selected algorithms in the single algorithm approach when predicting the three different problems. Balanced accuracy and NMSE are averaged across 100 cross-validation splits. Values in bold are significant on a 95% confidence level. BACC, balanced accuracy; NMSE, normalized mean squared error; SVM, support vector machine.

Table S3: Performance and confidence intervals of the best performing settings in the ensemble approach when predicting the three different problems. BACC and NMSE are averaged across 100 cross-validation splits. Values in bold are significant on a 95% confidence level. BACC, balanced accuracy; NMSE, normalized mean squared error.
