# Supplementary figures and images for: A machine-learning framework for robust and reliable prediction of short- and long-term treatment response in initially antipsychotic-naïve schizophrenia patients based on multimodal neuropsychiatric data
Source: Transl Psychiatry. 2020 Aug 10;10:276. doi: 10.1038/s41398-020-00962-8 (PMC7417553; doi:10.1038/s41398-020-00962-8)

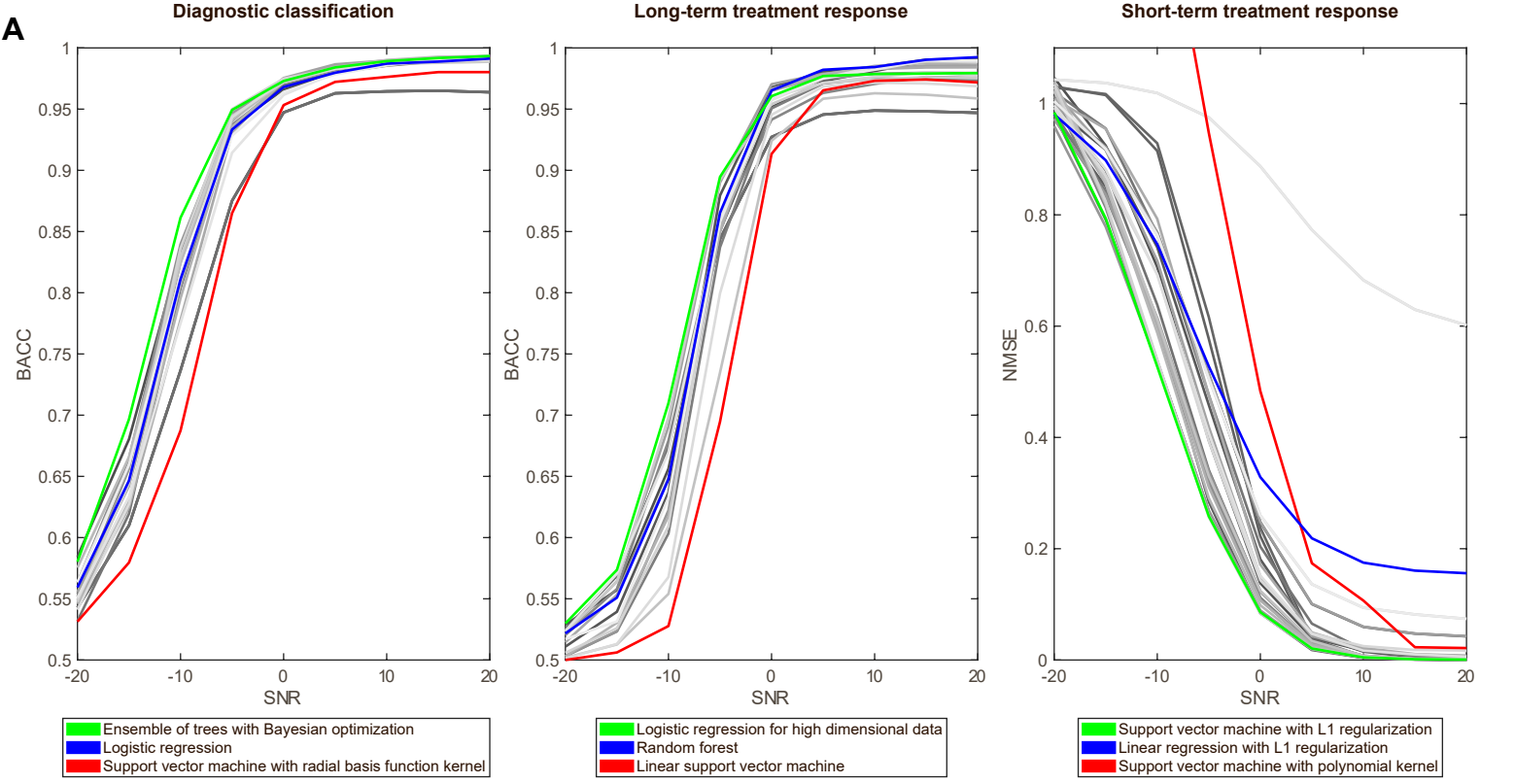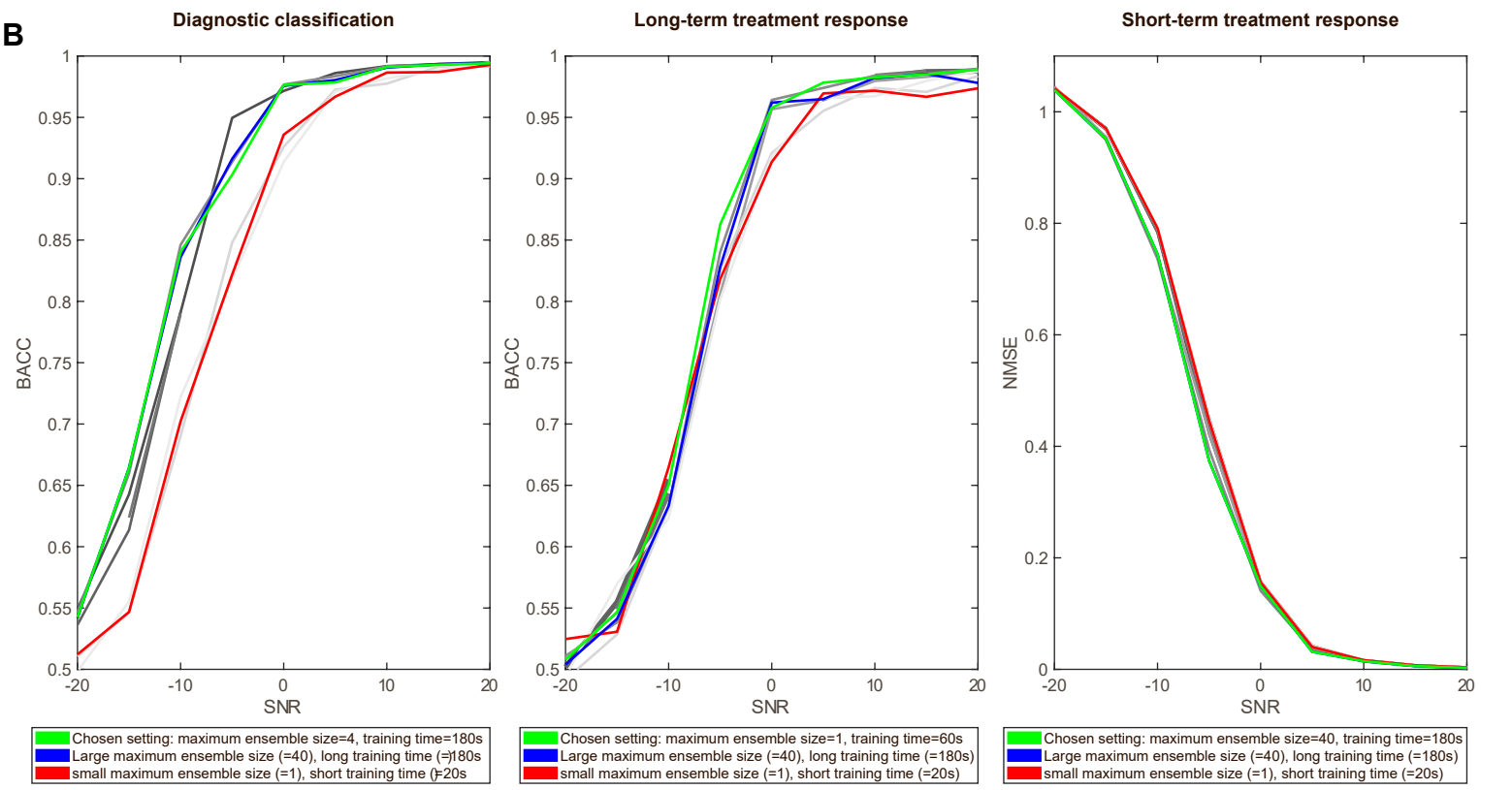

Supplement: Supplementary file 3 — Figure S1 [file 41398_2020_962_MOESM3_ESM.pdf]
